# Mycotoxins from *Alternaria Panax*, the specific plant pathogen of *Panax ginseng*

**DOI:** 10.1080/21501203.2023.2265662

**Published:** 2024-01-02

**Authors:** Huiqing Chen, Jianzi Liu, Ling Hu, Jian Yang, Yanduo Wang, Wensong Sun, Rong Wang, Gang Ding, Yong Li

**Affiliations:** a Key Laboratory of Bioactive Substances and Resources Utilization of Chinese Herbal Medicine, Ministry of Education, Institute of Medicinal Plant Development, Chinese Academy of Medical Sciences and Peking Union Medical College, Beijing, China; b Ningbo Academy of Inspection and Quarantine, Ningbo, China; c State Key Laboratory Breeding Base of Dao-di Herbs, National Resource Centre for Chinese Materia Medica, China Academy of Chinese Medical Sciences, Beijing, China; d Liaoning Research Institute of Cash Crops, Liaoyang, China

**Keywords:** *Alternaria panax*, *Panax ginseng*, mycotoxin, phytotoxic activities, dibutyl phthalate

## Abstract

Ginseng black spot, caused by *Alternaria panax*, is one of the most common diseases of *Panax ginseng*, which usually causes serious yield loss of ginseng plants. However, the pathogenic mechanism of *A. panax* has not been clarified clearly. Mycotoxins produced by phytopathogens play an important role in the process of infection. Previous study reported that dibutyl phthalate (DBP) identified from the metabolites of *A. panax* is a potent mycotoxin against *P. ginseng*. However, more evidence suggests that DBP is one of the constituents of plasticisers. To identify mycotoxins from *A. panax* and evaluate their phytotoxicity on the leaves of *P. ginseng*, different chromatographic, spectral and bioassay-guided methods were used together in this report. As a result, tyrosol (**1**), 3-hydroxy-3-(4-methoxyphenyl) propanoic acid (**2**), and 3-benzylpiperazine-2,5-dione (**3**) were isolated and characterised from the extract of *A. panax*, in which compounds **1** and **2** showed phytotoxic activity on ginseng leaves. Furthermore, DBP was confirmed to come from the residue of ethyl acetate through UPLC-MS/MS analysis, and displayed no phytotoxicity on ginseng leaves based on biological experiments. The results in this report first revealed that tyrosol (**1**), and 3-hydroxy-3-(4-methoxyphenyl) propanoic acid (**2**) not DBP were the potent mycotoxins of *A. panax*.

## 1. Introduction

The root of ginseng (*Panax ginseng*) has been used for thousands of years as one of the precious Traditional Chinese Medicines to bolster human immunity, provide nutrition, ameliorate fatigue, and enhance resistance in China and other Eastern Asian countries (Sticher 1998; Yeh et al. 2003). Since 2018, Chinese Pharmacopoeia allowed the root of *P. ginseng* to be added to food, which significantly expanded its consumer demand. Now, the global value of *P. ginseng* is approximately 2.0 to 3.5 billion dollars annually, as an important ginseng planting country, China contributes approximately 80% of the total yield of ginseng in the world (Hong et al. 2006; Baeg and So 2013; Dong et al. 2018). Ginseng production is mainly supplied by cultivation alternatives from China and other Eastern Asian countries, such as North Korea, South Korea, and Japan (Jung et al. 2003; Ying et al. 2012). As a perennial herbal medicine and food material, ginseng plants confronted the infection of various foliar and soilborne pathogens during their growing periods, and the final survival chance is no more than 25% without human intervention (Li et al. 2014; Dong et al. 2018).

So far, more than 10 important diseases have been reported on *P. ginseng*, some of which are destructive (Lee et al. 2011; Gao et al. 2014; Wang et al. 2015; Farh et al. 2018; Bischoff and Goodwin 2022). *Alternaria panax* is a host-specific fungus, which causes black spot symptoms on the above-ground parts of ginseng plants, especially on the leaves, stems, and fruits (Putnam and du Toit 2003; Li et al. 2019). This disease has been widely spread in the ginseng producing areas from China and other Asian countries since the 1980s, accounting for more than 20%–30% of incidence (Li et al. 2019), and leading to about 10%–20% yield loss annually (Chen et al. 2014; Zhang et al. 2016). Every year, when the environmental temperature is fittable, dormant conidia on ginseng residues begin to germinate and infect the stem of ginseng seedlings (Tao et al. 1992). Then, conidia from the developed black spots were taken as the primary infection source, finally resulting in serious black spots on the stem and leaves of adjacent ginseng plants through cycles of infections (Hirosawa and Takuda 1982). When ginseng seedlings are infected by *A. panax*, their stems would gradually girdle, then collapse, and finally damp off. For elder ginseng plants, foliar infections usually happen in summer by rapidly enlarging dark brown necrotic spots surrounded by chlorotic margins (Li et al. 2019).

It is well known that mycotoxins produced by phytopathogens of *Alternaria* genus play an important role in the process of infection, and many phytotoxic secondary metabolites were isolated from this genus (Fleck et al. 2012; Wei et al. 2017). In total, 17 groups of mycotoxins, including AF toxins, AAL toxins, AM toxins, tentoxins, etc., have been identified from metabolites of *Alternaria* species (Meena et al. 2017; Puntscher et al. 2018; Zhao et al. 2020). As for the ginseng black spots caused by *A. panax*, whether mycotoxins participated in the infection and the development of necrotic lesions was still unknown. Quayyum et al. (2003) reported that *A. panax* produces an AP protein toxin, but its advanced structure and amino acid sequence were not available, which made it difficult to carry out further research. Wang et al. (2014) reported that dibutyl phthalate (DBP) was the major mycotoxin produced by *A. panax*. Though there are several reports support that DBP and its analogues are secondary metabolites found in different plants and microorganisms, numerous researches reported that DBP and its analogues are the constituents of plasticisers (Di Bella et al. 1999; Guo et al. 2012; Sun et al. 2012). So, in the present work, we focus on the identification of mycotoxins in the extract of *A. panax*, and three secondary metabolites including tyrosol (**1**), 3-hydroxy-3-(4-methoxyphenyl) propanoic acid (**2**), and 3-benzylpiperazine-2,5-dione (**3**) were isolated, in which compounds **1** and **2** were revealed to possess phytotoxic effects on *A. panax* leaves. In addition, DBP was confirmed to originate from organic solvents based on and UPLC-MS/MS technique, and this chemical did not have phytotoxicity according to bioactive evaluation.

## 2. Materials and methods

### 2.1. General experimental apparatus

NMR data were acquired on a Bruker 500 spectrometer using solvent signal (CD_3_OD; *δ*_H_ 3.31) as reference. Sephadex LH-20 and silica gel were purchased from Pharmacia (Biotech, Sweden) and Shanghai Titan Scientific Co., Ltd. (Shanghai, China), respectively. Semi-preparative HPLC separation was performed on a Shimadzu LC-6AD instrument packed with a YMC-Pack ODS-A column. HR-ESI-MS spectra were analysed using an ESI-Q-TOF-MS (Waters Synapt G2, America). Ethyl acetate of analytical grade and ethanol absolute of analytical grade were purchased from Tianjin Beilian Fine Chemical Co., Ltd. (Tianjin, China). HPLC grade acetonitrile, methanol, water and formic acid were provided by Merck (Darmstadt, Germany). The standard of dibutyl phthalate (DBP) was purchased from J&K (Beijing, China).

### 2.2. General experimental procedures

The crude extracts from the liquid fermentation of *A. panax* were extracted by a KQ-500E Ultrasonic Cleaner (Ultrasonic Instrument Co., Ltd., Kunshan, China). The sample was concentrated under reduced pressure with a rotary evaporator device (EYELA, Tokyo, Japan), which was equipped with a low-temperature cooling circulation pump (Great Wall Scientific Industrial and Trade Co., Ltd., Zhengzhou, China) and a vacuum diaphragm pump (iLMVAC Co., Ltd., Germany). The crude extracts and standard were analysed by a UPLC-Q-TOF-MS/MS system (Waters, United States). Chromatographic analysis was carried out with a Waters ACQUITY UPLC-PDA system equipped with an analytical reverse-phase C-18 column (2.1 mm × 100 mm, 1.7 μm, ACQUITY BEH, Waters, United States) with an absorbance range of 200 nm to 400 nm. Time-of-flight MS detection was performed with a Xevo G2-SQTOF system (Waters), equipped with an electrospray ionisation source (ESI), and the mass data were obtained using Mass Lynx 4.1 software (Waters, USA).

### 2.3. Fungal strains

The pathogen causing black spot of *P. ginseng* has been identified as *A. panax* (Yu et al. 1984; Deng et al. 2012). The *A. panax* strain used in the present work was provided by Professor Junfan Fu from Shenyang Agricultural University in China, and the pathogenicity of it on *P. ginseng* has been confirmed (Wang et al. 1986a, 1986b).

### 2.4. Production, extraction, and purification of secondary metabolites

The fungus was grown on PDA plates at 25 °C for 7 days, then five mycelial cakes (8 mm in diameter) were taken from the edge of the colony, and inoculated into 300 mL sterilised PDB liquid culture, shaking cultured at 25 °C, 120 r/min for 14 days. The mycelium was separated from the culture filtrate by filtration through gauze. The aqueous filtrate (70 L) was then extracted with ethyl acetate three times (1/1, *v/v*) and the organic layer was evaporated in vacuo at 38 °C. Finally, 18.26 g of crude extract was obtained. The original extract was first fractionated on a silica gel column using di-chloromethane-methyl alcohol (1/0–0/1, *v/v*) progressively to give four fractions (F1–F4). The residue of F2 (857.4 mg) was further purified by silica gel column again using di-chloromethane-methyl alcohol (1:0–0:1, *v/v*) to give six subfractions (F2.1–F2.6). The residue of F2.1 was subjected to Sephadex LH-20 and eluted with MeOH to give fractions (F2.1.1–F2.1.4). F2.1.2 (28.1 mg) was separated by semi-preparative HPLC (0–35 min, 20–100% MeOH in water, 2 mL/min, 254 nm and 210 nm) to obtain tyrosol (**1**, 4.1 mg) and 3-hydroxy-3-(4-methoxyphenyl) propanoic acid (**2**, 0.8 mg). The residue of F2.6 (26.7 mg) was further purified by semi-preparative HPLC (0–35 min, 20–100% MeOH in water, 2 mL/min, 254 nm and 210 nm) to obtain 3-benzylpiperazine-2, 5-dione (**3**, 3.2 mg).

#### 2.4.1. Tyrosol (1)

White crystal, ^1^H-NMR [600 MHz, (CD_3_)_2_CO]: 8.12 (1 H, s, OH-4), 7.05 (2 H, m, H-2, 6), 6.74 (2 H, m, H-3, 5), 3.68 (2 H, td, *J* = 7.2, 6.6, 3.6 Hz, H-8), 3.61 (1 H, t, *J* = 5.4 Hz, OH-8), 2.70 (2 H, t, *J* = 7.2 Hz, H-7). ^13^C-NMR [150 MHz, (CD_3_)_2_CO]: 156.6 (C-4), 131.0 (C-1), 130.7 (C-2, C-6), 115.9 (C-3, C-5), 64.3 (C-8), 39.5 (C-7). These data were in agreement with those previously reported (Li et al. 2020); (+)-HR-ESI-MS: *m/z* = [M+H]^+^ = 139.0753 (calcd. 139.0759, C_8_H_10_O_2_).

#### 2.4.2. 3-hydroxy-3-(4-methoxyphenyl) propanoic acid (2)

White solid, ^1^H-NMR (600 MHz, CD_3_OD): 7.04 (m, 2 H, H-6, 10), 6.70 (m, 2 H, H-7, 9), 4.31 (1 H, dd, *J* = 7.8, 4.8 Hz, H-3), 3.69 (3 H, s, -OCH_3_), 2.95 (1 H, dd, *J* = 13.8, 4.8 Hz, H-2a), 2.83 (1 H, dd, *J* = 13.8, 7.8 Hz, H-2b). These data were in agreement with those previously reported (Bietti and Capone 2006).

#### 2.4.3. 3-benzylpiperazine-2, 5-dione (3)

Yellow solid, ^1^H-NMR (600 MHz, CD_3_OD): 7.31 (3 H, m, H-10, 11, 12), 7.22 (2 H, m, H-9, 13), 4.23 (1 H, td, *J* = 4.8, 3.6, 1.2 Hz, H-3), 3.42 (1 H, dd, *J* = 17.4, 1.2 Hz, H-6a), 3.24 (1 H, dd, *J* = 13.8, 4.8 Hz, H-7a), 2.99 (1 H, dd, *J* = 13.8, 4.8 Hz, H-7b), 2.62 (1 H, dd, *J* = 17.4, 1.2 Hz, H-6b). ^13^C-NMR (150 MHz, CD_3_OD): 170.2 (C-2), 168.8 (C-5), 136.5 (C-8), 131.6 (C-10, C-12), 129.8 (C-9, C-13), 128.6 (C-11), 57.6 (C-3), 44.8 (C-6), 41.0 (C-7). These data were in agreement with those previously reported (Coursindel et al. 2010); (+)-HR-ESI-MS: *m/z* = 205.0974 (calcd. 205.0977, C_11_H_13_N_2_O_2_).

### 2.5. Phytotoxic assays

Compounds **1**–**3** were tested on *Panax ginseng*, using the leaf infiltration assay at concentrations of 5 × 10^−3^ mol. The compounds were dissolved in 75% ethanol and then the solution was diluted with 75% ethanol to reach the required concentration. Fresh ginseng leaves were excised and sterilised with 75% ethanol and dried naturally. On the surface of the plant leaves, which were previously punctured with a sterile needle (two inoculating sites symmetrically distributed on a leaf), a droplet (20 μL) of compound solutions was applied over the area. The leaves were placed on the surface of a water-saturated filter paper in Petri dishes. Seventy-five percent ethanol was used as the control. The dishes were sealed with parafilm and incubated at 25 °C for 7 days in a temperature-regulated chamber in the dark. For each metabolite and plant species tested, three replications were performed.

## 3. Results

### 3.1. Phytotoxic activities of ethyl acetate (EtOac) crude extract from Alternaria panax

**Figure 1.**
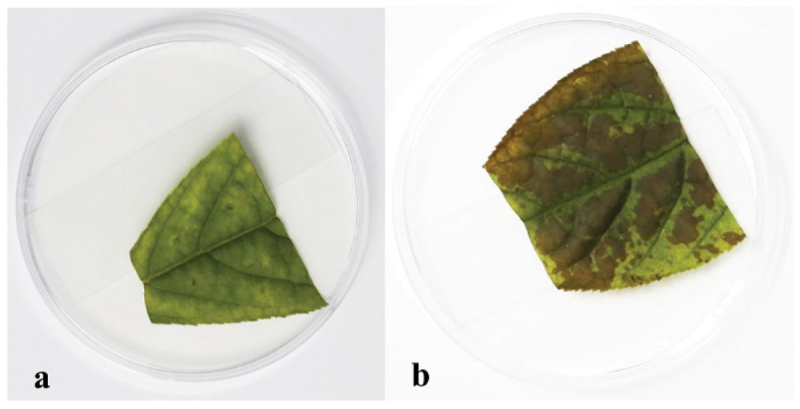
Phytotoxic activities of EtOAc crude extract against leaves of *panax ginseng*. 75% ethanol (control) (a), the EtOAc crude extract (b).

Many mycotoxins were isolated from *Alternaria* genus (Fleck et al. 2012; Wei et al. 2017). Thus, we speculated that *A. panax* could also produce potential mycotoxins with phytotoxic activities on its host plant *P. ginseng*. The phytotoxic activities of the EtOAc crude extract from the liquid culture of *A. panax* were evaluated against leaves of *P. ginseng*. After 7 days incubation at 25 °C in the dark, the colour of leaves inoculated with 75% ethanol in control had no obvious change (Figure 1a). However, the colour of leaves inoculated equivalent of EtOAc crude extract showed obvious chlorosis, and the colour of ginseng leaves was changed from green to dark brown with irregular lesions around the inoculating sites, and the lesions were gradually expanded to almost the whole leaves (Figure 1b). The symptom developed in the treatment of EtOAc crude extract was similar to those infected by pathogens in the field, which implied that some kinds of mycotoxins might be present in the metabolites of *A. panax*.

### 3.2. Bioassay-guided isolation of phytotoxic compounds

**Figure 2.**
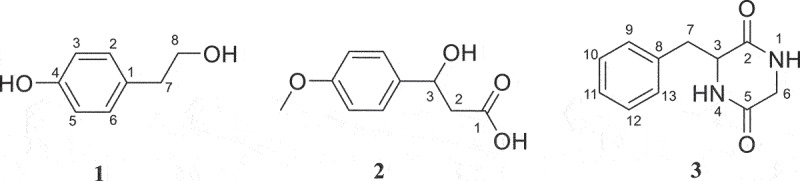
Structures of tyrosol (**1**), 3-hydroxy-3-(4-methoxyphenyl) propanoic acid (**2**), and 3-benzylpiperazine-2, 5-dione (**3**).

Different separation methods including silica gel column, Sephadex LH-20, and semi-preparative HPLC combined with the bioassay-guided process were used to isolate the potent phytotoxic metabolites from EtOAc extract of *A. panax*. Three metabolites (compounds 1–3, Figure 2) were finally purified through semi-preparative HPLC.

### 3.3. Structural analysis

**Figure 3.**
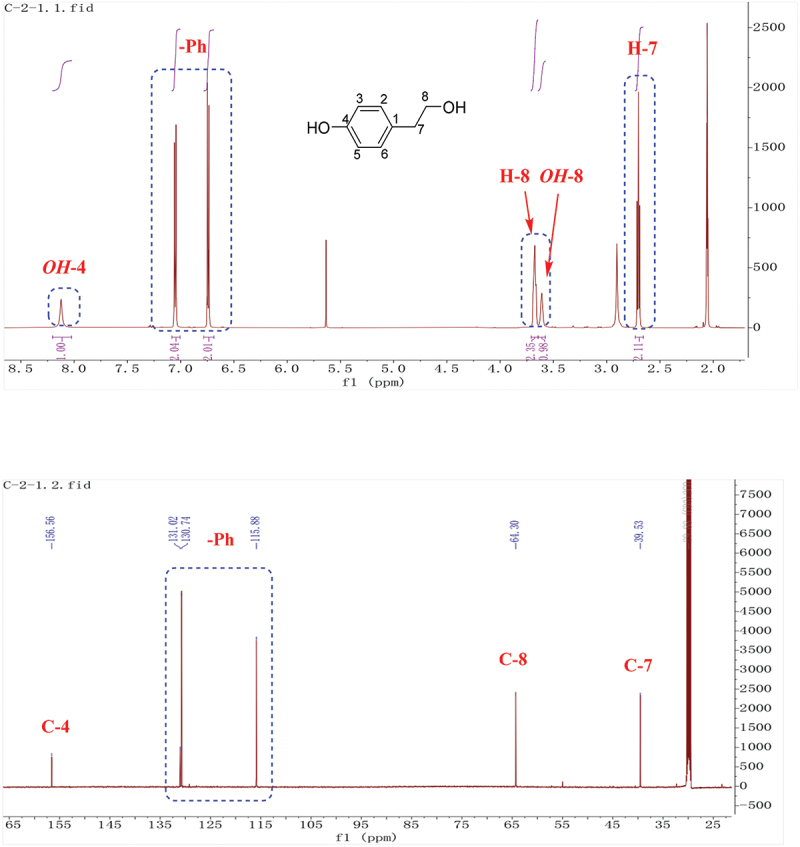
^1^H, ^13^C-NMR of compound **1**.

The molecular formula of **1** was determined to be C_8_H_10_O_2_ based on HR-ESI-MS. In the ^1^H, ^13^C-NMR spectrum of 1 [(CD_3_)_2_CO, Figure 3], there were four signals corresponding to aromatic hydrogens (*δ*_H_ 7.05, 2 H, m; *δ*_H_ 6.74, 2 H, m), of which coupling constants were both 8.4 Hz, indicating a *para*-substituted benzene ring. Two methylene signals (*δ*_H/C_ 3.68/64.3, 2 H, td, *J* = 7.2, 6.6, 3.6 Hz; *δ*_H/C_ 2.70/39.5, 2 H, t, *J* = 7.2 Hz) coupled to each other were also observed. Two free hydrogen signals at 8.12 and 3.61 ppm, respectively. Based on chemical shift values and coupling relationships, these two methylene were connected to the hydroxyl group (*δ*_H_ 3.61) and the benzene ring at C-1, respectively. The above data supported compound **1** as tyrosol (Li et al. 2020).

**Figure 4.**
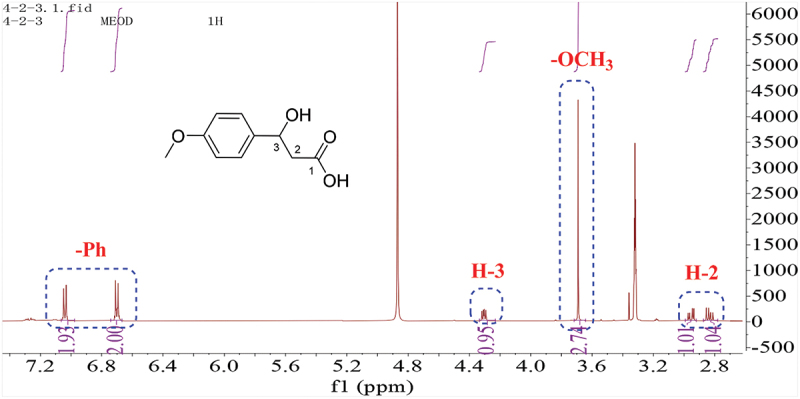
^1^H-NMR of compound **2**.

The ^1^H-NMR spectrum of **2** (CD_3_OD, Figure 4) revealed a *para*-substituted benzene ring. Four additional signals including one –OMe group at 3.69 ppm (3 H, s), one methine at 4.31 ppm (*J* = 7.8, 4.8 Hz), and one methylene group at 2.83 ppm (*J* = 13.8, 7.8 Hz) and 2.95 ppm (*J* = 13.8, 4.8 Hz) were present in the ^1^H-NMR spectrum, respectively (Figure 4), which were consistent with those of 3-hydroxy-3-(4-methoxyphenyl) propanoic acid (**2**) (Bietti and Capone 2006).

**Figure 5.**
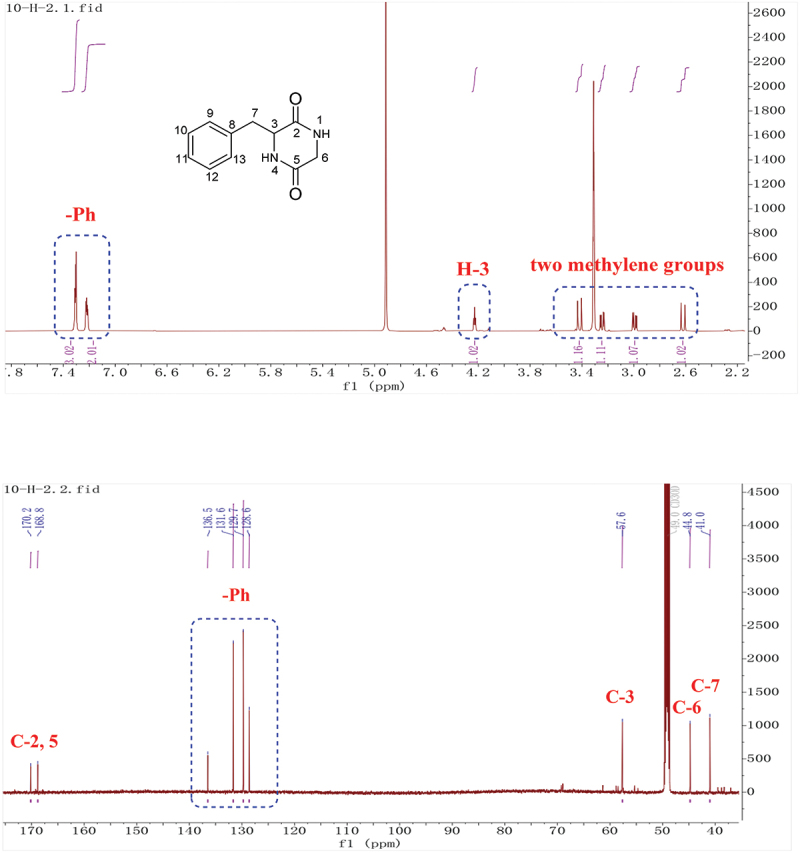
^1^H, ^13^C-NMR of compound **3**.

The molecular formula of **3** was established to be C_11_H_13_N_2_O_2_ based on HR-ESI-MS. The ^1^H and ^13^C-NMR spectra of **3** showed typical signals for a cyclopeptide. Two signals correspond to amide groups (*δ*_C_ 170.2, 168.8). According to ^1^H-NMR spectra, there was one nitrogen-bearing methylene group (*δ*_H/C_ 3.42, 2.62/44.8), one nitrogen-bearing methine (*δ*_H/C_ 4.23/57.6), five signals for a *mono*-substituted phenyl group (*δ*_H_ 7.31, 3 H, m; *δ*_H_ 7.22, 2 H, m) and one methylene group (*δ*_H_ 3.24, 2.99). Analysis of the chemical shift values and coupling constants (*δ*_H_ 2.99, *J* = 13.8, 4.8 Hz; 3.24, *J* = 13.8, 4.8 Hz; 4.23, *J* = 4.8, 3.6, 1.2 Hz) indicated the connection of C-3 and C-7 (Figure 5). The above data characterised compound **3** as 3-benzylpiperazine-2, 5-dione (Coursindel et al. 2010).

### 3.4. Phytotoxic activities of compounds 1–3

**Figure 6.**
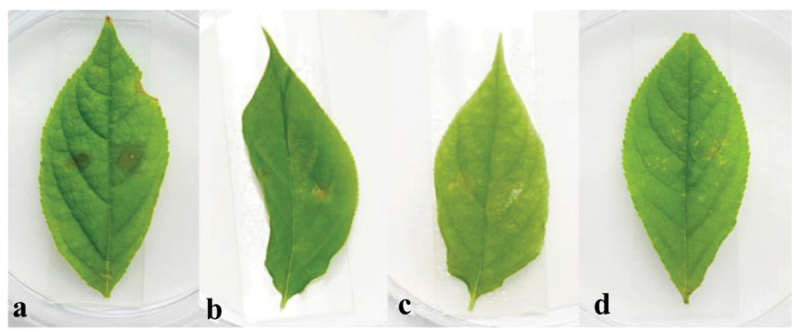
Phytotoxic activity of tyrosol (**1**) (a), 3-hydroxy-3-(4-methoxyphenyl) propanoic acid (**2**) (b), and 3-benzylpiperazine-2, 5-dione (**3**) (c) at 5 × 10^−3^ mol. 75% ethanol was used as the control (d).

The phytotoxic activities of compounds **1–3** were evaluated using the leaf infiltration assay in *P. ginseng* at the concentration of 5 × 10^−3^ mol. After 7 days incubation at 25 °C in the dark, leaves inoculated with 75% ethanol in the control have no obvious change (Figure 6d). However, in treatment inoculated compound 1, the leaves around the inoculating sites developed obvious water-soaking lesions, with the colour change from green to dark brown (Figure 6a). On ginseng leaves inoculated compound **2**, similar lesions were developed around the inoculating sites (Figure 6b), but the size of the lesions was not as big as those inoculated with compound **1**. As for compound **3**, no obvious lesions at the inoculating sites were observed (Figure 6c).

### 3.5. DBP was a plasticizing agent from the solvent EtOAc

**Figure 7.**
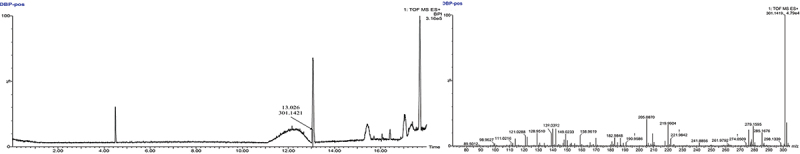
UPLC-ESI-TOF/MS/MS profiles of standard sample of DBP.

**Figure 8.**
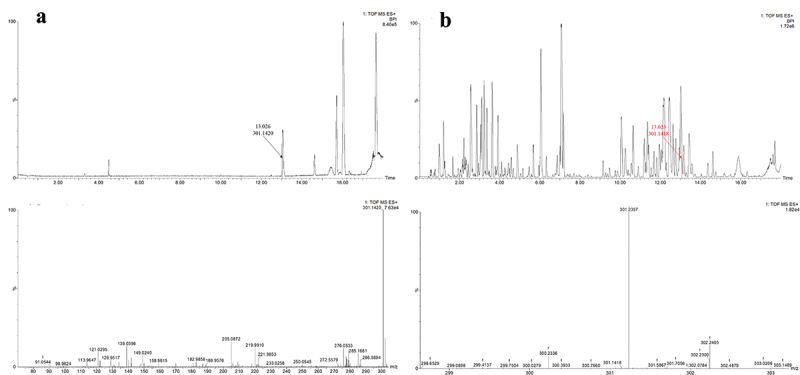
UPLC-MS/MS profiles of chemical pure EtOAc solvent (a); UPLC-MS/MS profiles of the EtOAc extract from liquid culture of *Alternaria panax* (b).

Wang et al. (2014) ever isolated DBP from the EtOAc extract of *A. panax*, and confirmed this compound as the potent mycotoxin of *A. panax*. The same method as that of Wang et al. (2014) was used in this report. When we analysed the constituents of the EtOAc extract based on UPLC-MS/MS experiment, DBP was found to exist in the extract (Figure 7). Then, chemical pure EtOAc solvent was analysed by the same procedure, and DBP was also found to be existed in the solvent based on its HR-ESI-MS and MS/MS analysis, which was the same as the data of the standard sample (Figures 7 and 8), implying that DBP was not a mycotoxin from *A. panax* but as a plasticising agent from the solvent EtOAc.

**Scheme 1.**
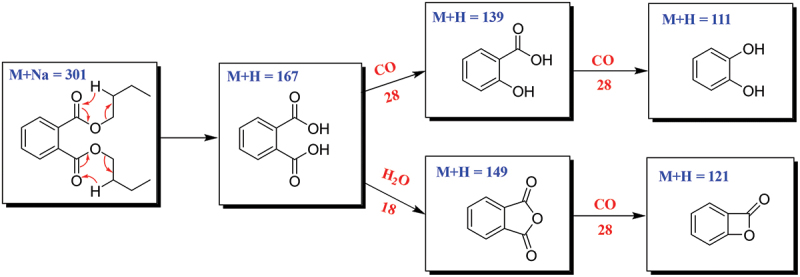
Possible fragmentation pathway of DBP.

The fragmentation pathways of DBP according to UPLC-Q-TOF-MS/MS analysis were depicted in Scheme 1, in which typical neutral loss and McLafferty rearrangement were the main fragmentation patterns for the parent ion *m/z* 301 [M+Na]^+^. The daughter ion (*m/z* 167) was formed from the parent ion (*m/z* 301) through McLafferty rearrangement by losing two molecules of CH_3_CH_2_CHCH_2_ (−56). Then, the daughter ion (*m/z* 167) had two fragmentation pathways, in which one formed the daughter ions *m/z* 139 and 111 by successively losing two molecules of CO (−28); the other lost one molecule of H_2_O (−18) to yield the daughter ion (*m/z* 149) which further lost a CO (−28) to produce the daughter ion (*m/z* 121). A large number of reports supported that DBP was one of the plasticiser constituents that existed in different organic solvents (Di Bella et al. 1999; Guo et al. 2012; Sun et al. 2012).

**Figure 9.**
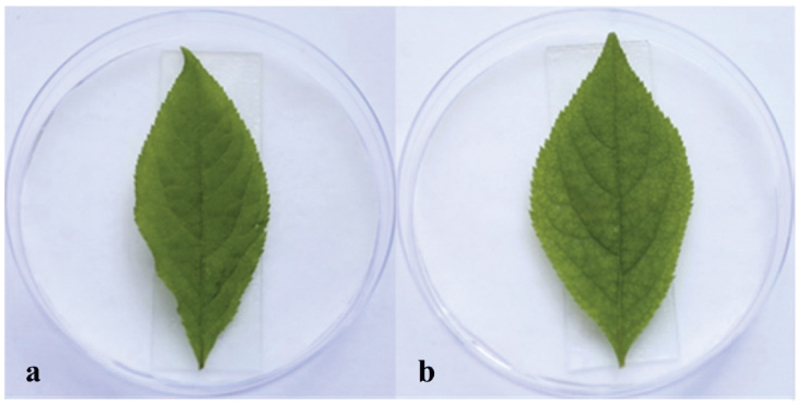
Leaf infiltration assay; 75% ethanol (control) (a), DBP (b).

Wang et al. (2014) reported that DBP has phytotoxic effect on ginseng leaves. The biological evaluation experiment of DBP on ginseng leaves was then investigated. Surprisingly, DBP did not display any phytotoxic activities against ginseng leaves compared with the positive control (Figure 9), which further confirmed that DBP was not the mycotoxin of *A. panax*.

## 4. Discussion

*A*. *panax* as the causal agent of the leaf black spot of *P. ginseng* could produce phytotoxic metabolites in liquid culture, which was confirmed by *in vitro* biological evaluation (leaf infiltration assay). Then, three compounds (**1–3**) were purified through a bioassay-guided approach, which were identified as tyrosol (**1**), 3-hydroxy-3-(4-methoxyphenyl) propanoic acid (**2**), and 3-benzylpiperazine-2,5-dione (**3**), respectively, based on NMR spectral data and comparison of the corresponding literatures. Tyrosol (**1**) showed obvious phytotoxicity on ginseng leaves. This secondary metabolite (**1**) is a known phytotoxin produced by various pathogenic fungi (Varejão et al. 2013; Di Lecce et al. 2021), such as the grapevine pathogenic fungi *Lasiodiplodia euphorbicola*, *L. hormozganensis* (Cimmino et al. 2017), *Neofusicoccum australe* (Masi et al. 2020), *N. luteum* (Reveglia et al. 2019), and *N. parvum* (Evidente et al. 2010). It also could be produced by *Diplodia seriata* (syn. *Botryosphaeria obtusa*), a pathogen of apple and frogeye leaf with phytotoxicity to tomatoes (Reveglia et al. 2019). Moreover, compound **1** was a quorum sensing molecule found in *Candida albicans* as well (Sebaa et al. 2019).

Compounds **2** and **3** showed weaker phytotoxicity to ginseng plants, from which we suspected that these two metabolites might be an accessory virulence factor. There were few phytotoxic reports about 3-hydroxy-3-(4-methoxyphenyl) propanoic acid (**2**), which was used more often as an intermediate in organic synthesis (Flowers et al. 2009; Parra et al. 2010; Koszelewski et al. 2015). 3-benzylpiperazine-2,5-dione [**3**, cyclo (Phe-Gly)] was a diketopiperazine first isolated from *A. panax* in this study. Diketopiperazines were a member of the mycotoxins of *Alternaria* genus (Meena et al. 2017). For example, maclosin [cyclo(L-Pro-L-Tyr)] is a host-specific phytotoxin, which can induce lesions on knapweed leaves at 10^−5^ (Stierle et al. 1988). Cyclo(Pro-Phe) isolated from *Alternaria alternata*, also showed phytotoxicity on spotted knapweed Sticher (1998. They shared the same skeleton and similar structures with compound **3**, suggesting that compound **3** may have potential phytotoxic activities on other plants as well. This metabolite (**3**) was isolated from *Theobroma cacao*, *Bacillus amyloliquefaciens*, *B. cereus* (Stark and Hofmann 2005; Kumar et al. 2014; Li et al. 2018 and endophytic fungus of *Kandelia candel* (Huang et al. 2007). It is one of the bitter tastes contributed compounds in *T. cacao* (Stark and Hofmann 2005) and has showed acaricidal and inhibitory activities against *Aspergillus* species (Stark and Hofmann 2005).

Wang et al. (2014) considered that DBP was a possible mycotoxin produced by *A. panax*. A few of studies also reported that DBP analogs were secondary metabolites from different plants/microorganisms (Zhao and Yang 2014; Li et al. 2015; Zhang et al. 2016). However, more evidences supported DBP is a plasticising agent. As early as 1940, DBP was considered to be one of the plasticisers (Fordyce and Meyer 2002; Vingiani et al. 2022). Feng et al. (2022) also showed that phthalates (DBP analogs) are primarily used in poly-vinyl chloride (PVC) plastics to increase product durability and flexibility. Furthermore, phthalates were confirmed to alter hormone levels and some chronic diseases (Hoppin et al. 2013; Whyatt et al. 2014; Hu et al. 2020), and phthalate metabolites were identified and classified by a nontargeted analysis approach (Feng et al. 2022). It is well-known that DBP can be easily found in organic solvents due to different ways. Thus, using organic solvents containing DBP to extract chemical materials such as fermentation broth, it is reasonable to find the potent residue of DBP in the extract.

## 5. Conclusion

In conclusion, three secondary metabolites including tyrosol (**1**), 3-hydroxy-3-(4-methoxyphenyl) propanoic acid (**2**), and 3-benzylpiperazine-2,5-dione (**3**) were isolated from the extract of *A. panax*, which were characterised based on NMR analysis. Compounds **1** and **2** displayed phytotoxic effects on ginseng leaves. Furthermore, DBP was confirmed to come from the residue of ethyl acetate according to UPLC-MS/MS analysis results. More importantly, DBP displayed no phytotoxicity on ginseng leaves based on biological experiments. The results in this report first revealed that tyrosol (**1**), and 3-hydroxy-3-(4-methoxyphenyl) propanoic acid (**2**) not DBP were the potent mycotoxins of *A. panax*, and the possible pathogenic mechanism of mycotoxins on ginseng leaves is ongoing in our lab.
